# Supplementary figures and images for: Hypnozoite depletion in successive Plasmodium vivax relapses
Source: PLoS Negl Trop Dis. 2022 Jul 22;16(7):e0010648. doi: 10.1371/journal.pntd.0010648 (PMC9348653; doi:10.1371/journal.pntd.0010648)

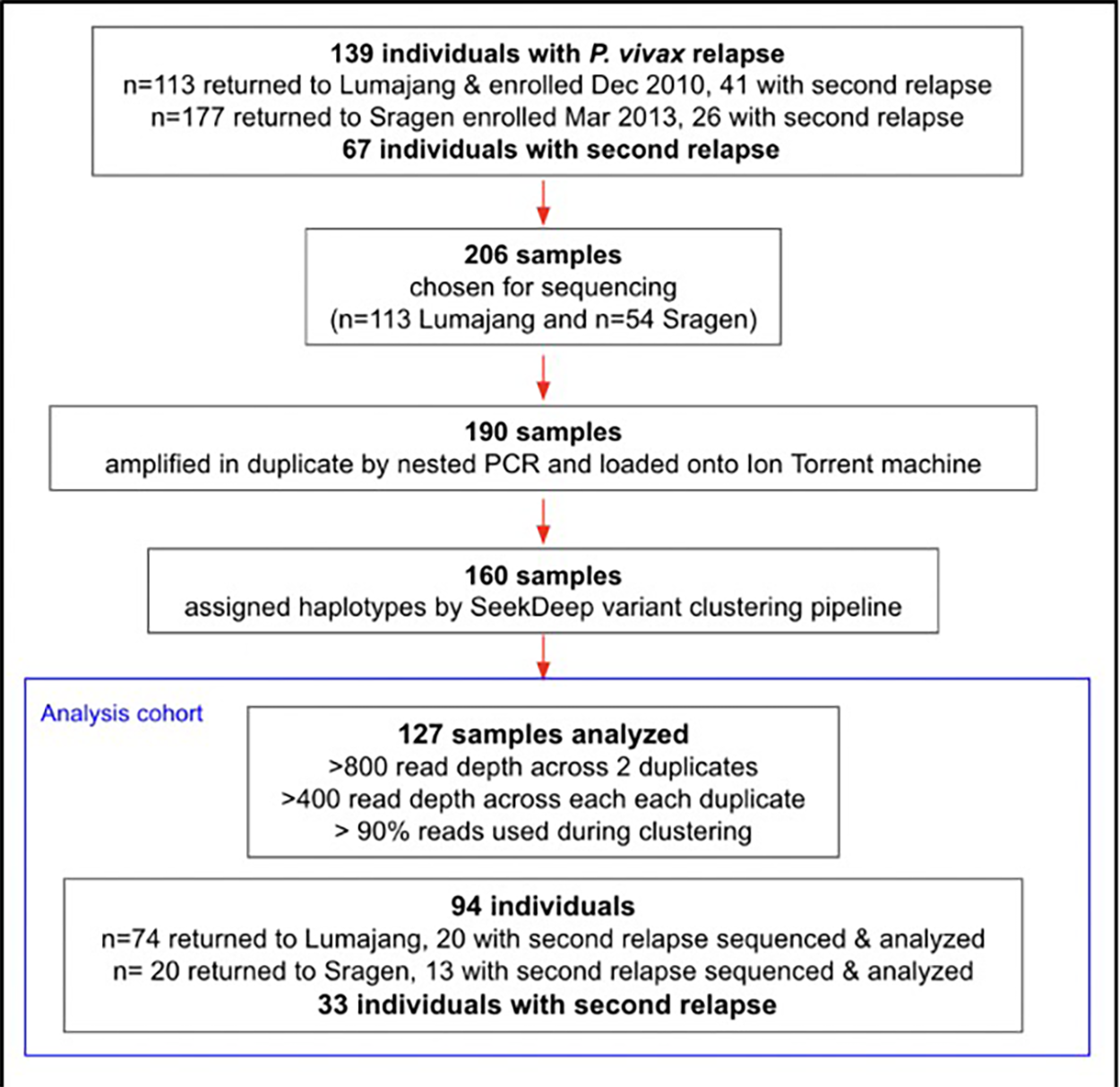

Supplement: S1 Fig — All samples available from the Lumajang cohort were selected for sequencing, while only samples from individuals with dual relapse were selected from the Sragen cohort. The final cohort used for sample analysis represented 62% (127/206) of the original sample set, with equal attrition from both cohorts. (TIF) [file pntd.0010648.s001.tif]

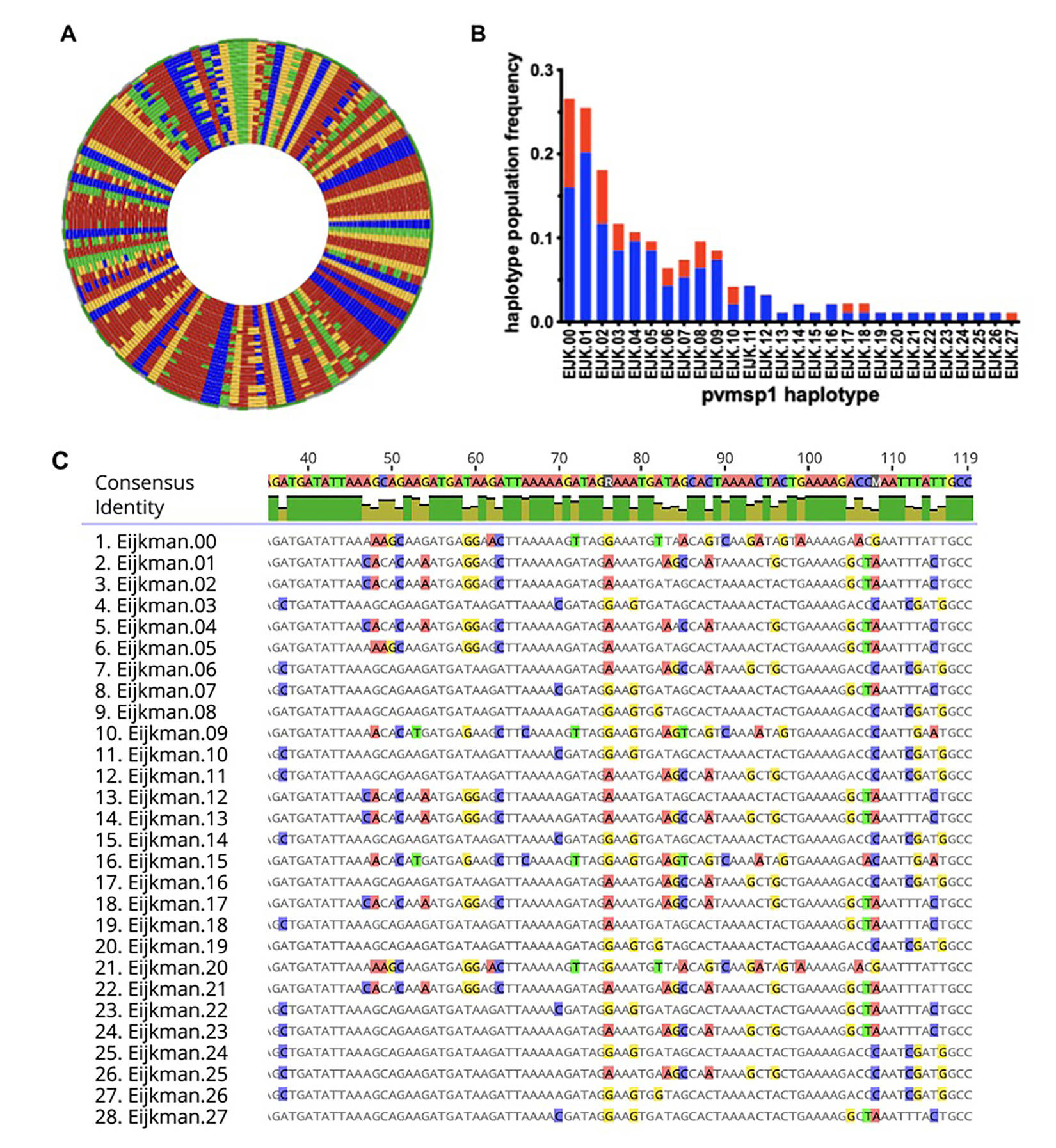

Supplement: S2 Fig — (A) DNA alignment of the 28 pvmsp1 haplotypes detected in the 127 samples from 94 individuals. Each concentric ring represents a unique sequence, differing by at least one single-nucleotide polymorphism at one of 44 variable sites detected within the 117 bp amplicon. Nucleotides are represented by different colors (adenine, red; thymine, blue; cytosine, green; and guanine, yellow). (B) Frequency of unique pvmsp1 haplotypes within the study population (out of 127 isolates). The red portions of the columns represent the proportion that occurred as a minority variant (existing at 1–20% frequency within the individual isolate). The three most common haplotypes were detected in at least 15% of all samples and 9 haplotypes were singletons, found in only one sample each. (C) Alignment of all 28 haplotypes of pvsmp1. (TIF) [file pntd.0010648.s002.tif]
