# Supplementary material for: Hypnozoite depletion in successive Plasmodium vivax relapses
Source: PLoS Negl Trop Dis. 2022 Jul 22;16(7):e0010648. doi: 10.1371/journal.pntd.0010648 (PMC9348653; doi:10.1371/journal.pntd.0010648)
Supplement: Attachment — Submitted filename: PLOS - Hypnozoite depletion - Reponses to Reviewers_v3.pdf [file pntd.0010648.s004.pdf]

June 28, 2022

PLOS Neglected Tropical Diseases

Dear Editor:

We would like to resubmit the attached article entitled "***Hypnozoite depletion in successive Plasmodium vivax relapses*** " to be considered for publication in *PLOS Neglected Tropical Diseases.* Below, in bold, is the text of the reviewer's responses from the previous submission with our associated responses and references to changes made to the manuscript.

This article has not been submitted to or accepted by any other publications. All authors have reviewed and approved the final product.

Thank you for your consideration of this work.

Sincerely,

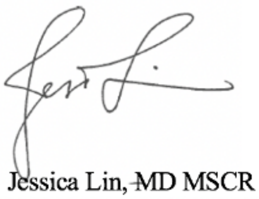

Jessica Lin, MD MSCR
Assistant Professor of Medicine
Institute of Global Health & Infectious Diseases
University of North Carolina - Chapel Hill

**Reviewer's Responses to Questions**

**Key Review Criteria Required for Acceptance?**
**As you describe the new analyses required for acceptance, please consider the following:**
**Methods**
**-Are the objectives of the study clearly articulated with a clear testable hypothesis stated?**
**-Is the study design appropriate to address the stated objectives?**
**-Is the population clearly described and appropriate for the hypothesis being tested?**
**-Is the sample size sufficient to ensure adequate power to address the hypothesis being tested?**
**-Were correct statistical analysis used to support conclusions?**
**-Are there concerns about ethical or regulatory requirements being met?**

**Reviewer #1: No further analyses likely to be helpful. Authors should list the actual numbers of the IRB approvals given in 2010 and 2013 such that the data can be linked to the specific approval documents.**
IRB approval numbers have been added to Methods section lines (93-94).

**Reviewer #2: Methods used mirrors similar studies, such as Chen et al 2007**

**While they only use one Allele (msp1) compared to the 3 used in Chen et al, the high res seq used compensates for this**
No changes needed.

**Reviewer #3: (No Response)**
--------------------
**Results**
**-Does the analysis presented match the analysis plan?**
**-Are the results clearly and completely presented?**
**-Are the figures (Tables, Images) of sufficient quality for clarity?**

**Reviewer #1: Analysis very similar to same authors 2015 JID paper from Cambodian parasites and appears to be sufficient.**

**Reviewer #2: Data presented sufficient, however would benefit form stratification if soldiers suffered acute vivax in Papua**
Unfortunately, acute malaria infections during deployment in Papua were not studied, though they would have been treated with dihydroartemisinin-piperaquine. This has been added to the Methods section (lines 82-83):

<u>"Unfortunately, the number and timing of acute vivax malaria attacks prior to disembarkment is not known; such attacks would have been treated with dihydroartemisinin-piperaquine."</u>

**Reviewer #3: (No Response)**

--------------------

**Conclusions**
**-Are the conclusions supported by the data presented?**
**-Are the limitations of analysis clearly described?**
**-Do the authors discuss how these data can be helpful to advance our understanding of the topic under study?**
**-Is public health relevance addressed?**

**Reviewer #1: Authors' conclusions are limited by the amount of data. They largely confirm their 2015 findings except the Indonesian soldiers with only one year's exposure had fewer relapses and less diverse parasites than those they observed in Cambodia.**

**Reviewer #2: conclusions are generally supported by the data, however it is important to have some clarity on the deployment infection rates**

While acute malaria cases among the deployed soldiers were not quantified, Ohrt et al. (reference 11) estimated the attack rate of Indonesian soldiers deployed in a nearby area of Papua to be 2 *P. vivax* cases/person-year. The mention of this statistic in the Methods section has been modified to clarify the significance of this attack rate (line 80):

"Of note, Papua, where their exposures occurred, has endemic *P. vivax* transmission with an attack rate among deployed Indonesian soldiers previously estimated at 2 vivax cases/person-year [11],"

**Reviewer #3: (No Response)**

--------------------

**Editorial and Data Presentation Modifications?**
**Use this section for editorial suggestions as well as relatively minor modifications of existing data that would enhance clarity. If the only modifications needed are minor and/or editorial, you may wish to recommend "Minor Revision" or "Accept".**

**Reviewer #1: (No Response)**

**Reviewer #2: (No Response)**

**Reviewer #3: (No Response)**

--------------------

**Summary and General Comments**
**Use this section to provide overall comments, discuss strengths/weaknesses of the study, novelty, significance, general execution and scholarship. You may also include additional comments for the author, including concerns about dual publication, research ethics, or publication ethics. If requesting major revision, please articulate the new experiments that are needed.**

**Reviewer #1: The manuscript repeats similar data reported by most of the same authors in JID 2015 (reference 13) from an endemic population in Cambodia. The current study's unique aspect is studying Indonesian soldiers who were exposed for a limited time period and then removed from the endemic area. As such, the general conclusion that there were fewer hypnozoites with less genetic variability fits the expected biology and is an important albeit unexciting observation.**

**I have a few questions that if the authors could answer and possibly add to the discussion would strengthen the manuscript.**

**There were two Indonesian battalions (Lumajang and Sragen) with very different (3:1) attack rates. Were there sufficient numbers to analyze the two groups separately as it would appear the former**

**group (presumably 2010) was more highly exposed to infection during their year in Papua? If so, this would suggest that MOI in the Sragen Battalion should be lower if it is truly a surrogate for hypnozoite number.**
The mean MOI of the first relapses (for individuals who experienced two relapses) was 1.9 among the Lumajang cohort (n=20) and 1.7 among the Sragen cohort (n=13); this difference was not statistically significant by a two-sample t-test (p=0.72). This calculation could only be performed on individuals with two relapses because the Sragen samples were only sequenced if the individual had two relapses, meaning the single-relapsers in the Lumajang group have no corresponding single-relapsers in the Sragen group. The mean number of days between enrollment and detection of the first relapse was 15 for Lumajang and 28 for Sragen (p=0.06). These differences are consistent with the hypothesis that a lower attack rate in the Sragen cohort correlates with lower MOI and longer latency until relapse, possibly due to lower hypnozoite burden in these less-exposed subjects, but our analysis lacks the sample size to detect statistically significant differences between these two groups.

**More than a decade has elapsed since these samples were obtained. Although genotyping is slow business, successive Indonesian battalions have since been enrolled in vivax relapse studies (e.g. tafenoquine). Are there any epidemiological data to support the authors' conclusion that infection / exposure is directly reflected in the number of hypnozoites / relapses observed in such units when removed from the endemic area?**

We do not have additional data beyond what is presented. We have not attempted similar genotyping in successive cohorts.

**The authors list a number of possible factors that might influence vivax relapses. One of the basic ones is hepatocyte senescence since the hypnozoites cannot exist outside of liver cell. As the average lifespan of hepatocytes is about 1 year, it would seem likely that older hypnozoites are presented with an 'activate or die' decision during the observation period that the authors were following the Indonesian soldiers. Was there any evidence of a 'burst' of activation at the end of the study period? Alternative suggestions as to explain why or how vivax infections eventually 'burn out'?**
We did not observe an increase in relapses near the end of follow-up (presumably near the end of a hepatocyte's life cycle). Rather, most relapses occurred early in follow-up, with few after 6 months. There is insufficient evidence to directly attribute this pattern to any of the mechanisms proposed, though it might be more consistent with hypnozoite senescence or immune clearance rather than hepatocyte turnover (which shouldn't change over the follow-up period).

**The authors generally conclude that the hypnozoites were activating independently while the alternative hypothesis is that successive malaria infections trigger the next relapse. Are these mutually compatible ideas on hypnozoite biology or do the authors just think that their population was different because they were no longer being exposed to current malaria infections?**
We cannot speak to the hypothesis of acute malaria provoking hypnozoite activation with this study population, either positively or negatively. Speculation that hypnozoites may reactivate independently

refers to the observation that most relapses are monoclonal despite the evidence that most infected subjects harbor multiple parasite clones; as such, most relapses occur from the reactivation of a single hypnozoite clone despite the possible presence of others. The existence of relapse triggers that influence hypnozoite reactivation cannot be ascertained from this data.

**Reviewer #2: This is an important set of data that further contributes to our understanding of the relapse biology of P. vivax infections. The power of this study is due to its use of a relatively homogenous 'non-immune' cohort (presumably mostly 18-30yrs male, healthy (this info was not given)) with a limited exposure to P. vivax infection (~1 year in Papua) before returning home to a non-endemic setting (Java). The high degree of homologous relapse and the postulated arguments for why this occurs make sense.**
**I would be grateful if the authors considered replying to the following questions and concerns**

**Major question: Did the soldiers receive any prophylactic treatment in Papua (i.e. Doxy?) and if not; did they have any acute infections in Papua (especially given the extended period of deployment one would expect at least one infection). and if so, what was used to treat the acute episode. This type of information is important as this type of data may be necessary to stratify the time to relapse data (as Chen 2007 has previously reported)**
The soldiers do not receive chemoprophylaxis in Papua. Indonesian army medical doctrine is to manage malaria with personal protection (bed nets, etc.) and diagnosis and treatment of the infected rather than chemoprevention. Those attacks certainly occurred and were managed with a curative regimen of dihydroartemisinin-piperaquine (according national guidelines). Unfortunately, we do not have this information on who suffered acute attacks prior to disembarkment and when.

We have added this information to the Methods (Lines 77-83 under "Study Population"):

"Study participants were Indonesian soldiers returning to Java (either Lumajang or Sragen) after being stationed in Papua, Indonesia for roughly one year. <u>Subjects did not receive malaria prophylaxis during their deployment as per Indonesian medical army doctrine, but received routine health screening upon arriving in Java and were treated with dihydroartemisinin-piperaquine if found to be malaria-positive by microscopy.</u> Of note, Papua, where their exposures occurred, has endemic *P. vivax* transmission with an attack rate previously estimated at 2 vivax cases/person-year [11], while Java no longer has endemic malaria transmission, excepting a few well known persisting foci [12]. <u>Unfortunately, the number and timing of acute vivax malaria attacks prior to disembarkment is not known; such attacks would have been treated with dihydroartemisinin-piperaquine</u>."

**Minor comments:**

**1. It would be good to describe the cohort (i.e sex, age)**

Added to the Methods section (75-76):

“Study participants were male Indonesian soldiers, aged 21-50 years old, returning to Java (either Lumajang or Sragen) after being stationed in Papua, Indonesia for one year.”

**2. Was any consideration given to using multiple alleles (or even a Microsat marker)rather than just one (msp) to give more confidence of clonality?**

All the PCR and sequencing work for this study was done in Indonesia, so we tried to choose a simpler workflow. Also, when the sequencing was originally completed (~2015-16), other hypervariable loci were not well-established as suitable targets for amplicon sequencing. We previously compared this msp1 amplicon approach with a combination of 3 microsatellite markers (Lin, JID 2015, Ref 13) and found that the one amplicon detected a higher multiplicity of infection. While a larger set of microsatellite markers might provide more power, this would’ve been much more work, and interpretation of phased haplotypes is impossible from such data. We already cite the single amplicon sequencing approach as a limitation in the Discussion (lines 211-216).

Even so, the finding that multiplicity of infection decreases over time is likely to stand given that the sensitivity of *msp1* sequencing to identify unique parasite clones should not have changed over the duration of the study period.

**Reviewer #3: Major comments**

**The authors applied targeted amplicon deep sequencing to study the genetics of relapsing P. vivax parasites among Indonesian soldiers returning from malaria areas after a 12-month deployment. The study provides new insights into the biology of hypnozoite activation causing relapses. The most interesting finding is the decreasing multiplicity of infection over time, consistent with hypnozoite depletion resulting from activation and other mechanisms. Although expected, this is the first report from patients. The manuscript is well written.**

**One weakness of the study is the use of single amplicon deep sequencing. The authors listed this as one of the limitations of the study and commented that this may limit the power of detecting minor variants and underestimate polyclonality. They then argued that given the genetic diversity revealed at pvmsp1, this difference would be incremental and not change overall findings. However, the level of genetic diversity revealed at pvmsp1 was relatively low with only 28 haplotypes detected from a set of 127 samples tested. This is in contrast to high genetic diversity levels reported from sample sets obtained from people living in high transmission settings where more haplotypes than sample numbers tested are commonly seen, especially in P. vivax infections. I assume the authors’ notion of high genetic diversity in this set of relapsing samples was based on the HE value of 0.77. However, this HE value does not seem to be correct based on 28 unique haplotypes obtained from 127 samples tested. Could the authors please describe how the HE value of 0.77 was derived?**

The HE value of 0.77 was derived from the mathematical equation found in reference 15, where virtual (or expected) heterozygosity was defined as $[n/(n-1)][1-\Sigma p_i^2]$, where n is the total number of isolates and $p_i$ is the population frequency of the *i*-th *allele*.

> [illegible]
> values. Virtual heterozygosity is defined as $H_E = [n/(n - 1)][1- \sum p_i^2]$, where n is the number of isolates analyzed and $p_i$ is the frequency of the *i*-th allele in the population. Values range between 0-1. Virtual heterozygosity, in this context, gives the average probability that a pair of alleles randomly obtained from the population is different. It is synonymous with the Simpson's index of diversity, which has previously been suggested by Hunter and Gaston (1988) as a means to evaluate the discriminatory ability of genotyping systems for bacterial strains. We [illegible]

To clarify, this value was calculated from the first detected relapses of 94 individuals (n=94), not from each of the 127 total successfully-sequenced isolates. This was to avoid double-counting alleles that appeared in dual relapses. Additionally, our data allows us to count multiple alleles that were detected in single sample. The following has been added to the results section (line 130):
**"**Expected heterozygosity ($H_E$) was high using a single marker ($H_E$=0.77 <u>among 94 first-relapse samples</u>, reflecting an average 77% probability that 2 samples taken at random from the population will display different *pvmsp1* haplotypes)."

**As the study participants were randomized into primaquine and no primaquine arms, it would be interesting to compare HE and MOI values between the two arms at 1st and 2nd relapses. This may reveal any difference in hyponozoite load between the two groups.**
Figure 2B shows the change in MOI over time between the primaquine and no primaquine groups. As noted in the results (lines 153-162), the change in MOI was greater among those treated with primaquine, likely due to the hypnozoiticidal effect of primaquine therapy. There was also a trend towards decreasing MOI over time in the non-primaquine arm, though this trend was only statistically significant in the similar, non-primaquine-treated Cambodian study. However, comparison between 1st relapses and 2nd relapses across arms is difficult because these relapse number designations are arbitrarily determined from the start of enrollment; the 2nd relapse in one subject may actually be their 3rd total relapse (with the first occurring before enrollment), and the 1st relapse of another subject may actually be their 4th. As such, this makes comparing across 1st relapses difficult, although trends noted within a given individual across their multiple relapses are still valid.

**Minor Comments**

**1. Abstract: "targeting amplicon sequencing" should be "targeted amplicon sequencing".**
Change made to Abstract (line 26)

**2. Introduction- 2nd paragraph: "burden of P. vivax in Southeast Asia [3,4]" should be changed to "Southeast Asia and South Pacific" as one of the references reports findings from PNG.**

Change made to introduction section lines (57-58).

**3. Results-Clonal patterns of relapsing P. vivax: "just 24% (8/33) of individuals had only one haplotype detected across both of their relapses, while 76% (21/33) had multiple haplotypes present across both relapses." It would be clearer to change this to "just 24% (8/33) of individuals had the same haplotype detected across both of their relapses, while 76% (21/33) had different haplotypes between two relapses."**

While this may be shorter, this could be misinterpreted as meaning "24% of individuals had the same haplotype found in both relapses, even if one or both relapses were polyclonal". Our original wording is meant to emphasize that 24% of individuals had two monoclonal relapses with the same haplotype found in both relapses, while the remaining 76% had at least two haplotypes detected across both of their relapses.

**4. Discussion: the first interesting observation of the high proportion of monoclonal parasites in relapse samples indicates individual hypnozoite clones activate independently of other clones present in the sample individual. This finding is consistent with findings of reference 8. It will strengthen the discussions by comparing the study findings and similarities between the two populations.**

Thank you for this suggestion. We have placed our findings in the context of the previous important study by adding several sentences to the 2nd paragraph of the Discussion (lines 183-190):

"We believe this likely reflects lower hypnozoite loads in our cohort, akin to the Australian. soldiers previously described to have suffered monoclonal relapses (85/86 relapse isolates) after deployment in East Timor, in contrast to the East Timorese resident population who were more likely to harbor polyclonal infection [8]. When multiple relapses were observed in the Australian soldier cohort, they too more frequently showed heterologous genotypes. The major difference was the receipt of prophylaxis both during deployment and terminal prophylaxis at the end of deployment, which likely led to the much longer time interval to relapse (median 181 days vs. 30 days in the Indonesian soldiers who did not receive chemoprophylaxis). The less frequent polyclonal relapses in these returning soldier cohorts could also be due to the absence of hypothesized environmental triggers for hypnozoite activation related to malaria exposure (e.g. infective vector bites or malaria superinfection), reducing the chance that multiple clones will reactivate simultaneously."

--------------------

**PLOS authors have the option to publish the peer review history of their article. If published, this will include your full peer review and any attached files.**

**If you choose "no", your identity will remain anonymous but your review may still be made public.**

**Do you want your identity to be public for this peer review?**

**Reviewer #1: No**

**Reviewer #2: No**

**Reviewer #3: No**
